# An Early Victim

**Published:** 1891-05-02

**Authors:** 


### Passing Topics.

### AN EARLY VICTIM.

It was but a month ago we commented upon certain statements put forward in regard to the effect of the occupation of nursing upon the duration of life, and we ventured to assert there was no ground for believing that nurses, as a body, when they entered upon their work, accepted the certainty of a shortened existence.

It is so frequently the case that publicity is given to matters concerning our nurses that we need not wonder at the paragraph which has been going the round of the papers in reference to the lamented death of Lady Alexandra Leveson-Gower. Even in those times, not yet left very far behind, when the craze of nursing was at its height, the daughters and nieces of dukes were necessarily few and far between in the ranks, and it would be useless to complain, as it would be to deny that, constituted as human nature is, the early death of this lady claims a more conspicuous notice and attracts more than ordinary comment because of her high social position.

All the more necessary, as it seems to us, it is that one statement to which public utterance is given should be accepted with caution. It is nothing less than that Lady Alexandra's death is attributable to the fact that she was required to perform the hard and menial duties of a housemaid and a scrubber. Some of us might be disposed to think that even if the charge were true many of the other duties required of a nurse would be infinitely more trying to a refined and delicate organisation, but letting that pass, we may remark without, as we believe, fear of contradiction, that there is no metropolitan hospital of repute at which nurses are required to scrub, or to perform duties which fairly and without qualification could be described as the duties of a housemaid.

Of course, it goes without saying that a nurse's duties comprise a considerable amount of hard and unpleasant work. The constant standing and walking, the multifarious attentions to be rendered to the patients, involving lifting and other similar demands upon muscular strength, with other duties which need not be specifically stated, and the constant strain upon the attention, go to make up a day's work at which no doubt many of the domestic servant class of persons would stand dismayed. No one can deny that a nurse has plenty to do, but we must enter a protest against the nonsense uninformed persons are frequently disposed to talk, and which we dare say most sensible nurses are as ready as anyone to resent.

It is not long ago since some amusement was caused by the attempt to denounce the management of a hospital because the night nurses were expected to empty, rinse, and refill the ward inkstands and to trim the lamps in use for medical purposes. In how many middle-class houses, we wonder, would not the lady prefer to trim her own lamps? We know pretty well that the opinion of many householders who have made use of the services of trained nurses in their homes is that the nurses want a great deal too much waiting upon as it is. What will it be when they are encouraged to demand that every duty, which by any stretch of fancy can be termed "menial," is to be taken from them?

Given good health and plenty of strength, without which no woman ought to think of becoming a nurse, and there is nothing in the actual work likely to hurt her. The wear and tear is less muscular than mental, and any bodily injury she may suffer is more likely to be referable to the atmospheric and other similar conditions than to an excess of actual labour. Poor Lady Alexandra, highly placed and accustomed to luxury and soft living, was scarcely likely to bear the strain of training, and the true lesson to be learned from her early death is rather the desirability of ensuring that none but really fit persons shall be entered as probationers than the necessity for eliminating all muscular work, which is not improbably the most healthful, if the least acceptable, of the several duties required at a nurse's hands.—Agricola.

